# Conversion of a molecular classifier obtained by gene expression profiling into a classifier based on real-time PCR: a prognosis predictor for gliomas

**DOI:** 10.1186/1755-8794-3-52

**Published:** 2010-11-10

**Authors:** Satoru Kawarazaki, Kazuya Taniguchi, Mitsuaki Shirahata, Yoji Kukita, Manabu Kanemoto, Nobuhiro Mikuni, Nobuo Hashimoto, Susumu Miyamoto, Jun A Takahashi, Kikuya Kato

**Affiliations:** 1 Research Institute, Osaka Medical Center for Cancer and Cardiovascular Diseases, 1-3-3 Nakamichi, Higashinari-ku, Osaka, 537-8511, Japan; 2 Department of Neurosurgery, Kyoto University Graduate School of Medicine, 54 Kawahara-cho, Shogoin, Sakyo-ku, Kyoto-shi, Kyoto, 606-8507, Japan; 3 National Cerebral and Cardiovascular Center, 5-7-1 Fujishiro-dai, Suita, Osaka 565-8565, Japan; 4 Kitano Hospital. 2-4-20 Ohgimachi, Kita-ku, Osaka, 530-8480, Japan

## Abstract

**Background:**

The advent of gene expression profiling was expected to dramatically improve cancer diagnosis. However, despite intensive efforts and several successful examples, the development of profile-based diagnostic systems remains a difficult task. In the present work, we established a method to convert molecular classifiers based on adaptor-tagged competitive PCR (ATAC-PCR) (with a data format that is similar to that of microarrays) into classifiers based on real-time PCR.

**Methods:**

Previously, we constructed a prognosis predictor for glioma using gene expression data obtained by ATAC-PCR, a high-throughput reverse-transcription PCR technique. The analysis of gene expression data obtained by ATAC-PCR is similar to the analysis of data from two-colour microarrays. The prognosis predictor was a linear classifier based on the first principal component (PC1) score, a weighted summation of the expression values of 58 genes. In the present study, we employed the delta-delta Ct method for measurement by real-time PCR. The predictor was converted to a Ct value-based predictor using linear regression.

**Results:**

We selected *UBL5 *as the reference gene from the group of genes with expression patterns that were most similar to the median expression level from the previous profiling study. The number of diagnostic genes was reduced to 27 without affecting the performance of the prognosis predictor. PC1 scores calculated from the data obtained by real-time PCR showed a high linear correlation (r = 0.94) with those obtained by ATAC-PCR. The correlation for individual gene expression patterns (r = 0.43 to 0.91) was smaller than for PC1 scores, suggesting that errors of measurement were likely cancelled out during the weighted summation of the expression values. The classification of a test set (n = 36) by the new predictor was more accurate than histopathological diagnosis (log rank p-values, 0.023 and 0.137, respectively) for predicting prognosis.

**Conclusion:**

We successfully converted a molecular classifier obtained by ATAC-PCR into a Ct value-based predictor. Our conversion procedure should also be applicable to linear classifiers obtained from microarray data. Because errors in measurement are likely to be cancelled out during the calculation, the conversion of individual gene expression is not an appropriate procedure. The predictor for gliomas is still in the preliminary stages of development and needs analytical clinical validation and clinical utility studies.

## Background

Since the inception of gene expression profiling, researchers have sought to use this technology to improve the diagnosis of diseases, especially cancers. Recently, MammaPrint [1,2] and Oncotype DX [3,4] were established as diagnostic tests based on multiple gene assays for breast cancer. Despite the success of these diagnostic tests, the development of assays for gene expression profiling is still difficult. In particular, there have been few examples of microarray-based diagnostic tests, although microarrays are frequently used as a discovery tool. One reason for the paucity of microarray-based diagnostic tests is that DNA microarrays require considerable effort to achieve the level of technical refinement necessary for diagnostic practice. On the contrary, real-time PCR is stable and robust and is frequently used for diagnosis. Because there are many studies describing the use of microarrays at the discovery phase, a convenient method to convert a microarray-based algorithm into one based on real-time PCR would help to accelerate the development of diagnostic systems based on gene expression profiling.

Previously, we performed gene expression profiling of 152 glioma tissues [5] with a high-throughput quantitative PCR technique called adaptor-tagged competitive PCR (ATAC-PCR) [6,7]. ATAC-PCR is an advanced version of quantitative competitive PCR characterised by the addition of unique adaptors for different cDNAs. A single ATAC-PCR reaction includes five cDNA samples and two different amounts of a control cDNA sample with different adaptor tags, and it measures the relative expression of the samples against that of the control. We discovered a correlation between gene expression profiles and glioma prognosis, and we developed a prognosis predictor based on a 58-gene profile [5]. The performance of the predictor based on ATAC-PCR was cross-validated with a learning set of 110 glioma samples and validated with a test set of 42 samples. Cox regression analysis revealed that the correlation between the predictor and the prognosis was superior to that of histological classification and was an independent risk factor. The current prognostic standard, the histopathological classification system, is limited in its diagnostic accuracy, and prognoses range widely even within the same grade. Diagnosis depends on individual pathologists, and the results are often discordant among multiple pathologists [8]. The performance of the prognosis predictor based on ATAC-PCR indicated that this predictor held promise for the support of conventional histopathological classification. Our classifier is also expected to bring benefits in the clinical setting for personalized management of glioma patients. For example, various molecular-targeted drugs have recently been evaluated in clinical trials for gliomas. These novel treatments should be considered for tumours that are resistant to conventional chemoradiotherapy. Yet, it is important to avoid using such a therapy for tumours that are sensitive to conventional chemoradiotherapy, based on the cost and adverse effects associated with this technique. Considering elevated expression of angiogenesis-related genes in the poor prognosis group, [5], our classifier might be useful for selection of patients for anti-VGEF agents.

In the present study, we converted the conventional predictor to one based on real-time PCR. This new predictor is based on the delta-delta Ct method [9] and requires only the measurement of the cycle threshold (Ct) of diagnostic genes. For the conversion, we first identified a reference gene for real-time PCR. Then we constructed the parameters for the conversion formula using data obtained from the learning set, which was used to construct the original classifier. Finally, the new classifier was validated with a test set. Because there is a linear correlation between microarray data and Ct values [10], the conversion process could be applicable for classifiers based on microarrays.

## Methods

### Patients and tumour samples

Specimens excised from 80 patients with high-grade glioma (69 cases of glioblastoma and 11 cases of anaplastic astrocytoma) at Kyoto University Hospital or nearby regional hospitals between 1998 and 2008 were stored at -70°C until use. All histological diagnoses were performed in the Kyoto University Pathology Unit according to the 2000 or 2007 WHO classifications.

Sixty of the 80 samples were recruited from those used in the previous study [5]. They were collected from patients enrolled in a phase II clinical trial using nimustine, carboplatin, vincristine, and IFN-β with radiotherapy for high-grade gliomas (the KNOG study) [11]. The remaining 20 patients were treated with temozolomide and radiotherapy. The learning set included 44 samples (43 glioblastoma, 1 anaplastic astrocytoma) from the KNOG study. Recurrence was detected in 36 of the 44 patients and their median progression-free survival was 7 months. The test set included 36 samples (26 glioblastoma and 10 astrocytoma). Twenty-three of the 36 patients showed tumour progression, and their median progression-free survival was 8 months.

Institutional approval for this study was obtained from the Institutional Review Board of Kyoto University, and informed consent was obtained from all patients prior to surgery.

### RNA extraction and cDNA synthesis

Total RNA was isolated from 100 mg of the tumour specimen using TRIzol (Invitrogen, Carlsbad, CA, USA) according to the manufacturer's instructions. RNA concentrations and A260/A280 ratios were measured using a NanoDrop ND-1000 (NanoDrop Technologies, Montchanin, DE, USA). Only RNA samples with A260/A280 ratios above 1.90 were included in the study. RNA integrity was confirmed by analysis with the Agilent 2100 bioanalyser.

After DNase treatment, 5 μg of total RNA in 10 μl of distilled water was incubated with 1 μl of oligo(dT) primer for 5 min at 70°C. Total RNA was reverse transcribed in a total volume of 20 μl containing 4 μl of 5× first strand buffer, 1 μl of RNase inhibitor (Invitrogen), 2 μl of 0.1 M DTT, 0.5 μl of 20 mM dNTP and 1 μl of SuperScript III Reverse Transcriptase (Invitrogen). The samples were incubated at 45°C for 1 hr. Next, a reaction mixture (total volume of 103 μl) containing 10 μl of 10× *Escherichia coli *(*E. coli*) ligation buffer, 2 μl of 20 mM dNTPs, 2 μl of 0.1 M DTT, 2 μl of *E. coli *ligase (Invitrogen), 1 μl of RNase H (Invitrogen), 4 μl of *E. coli *DNA polymerase (Invitrogen) and 82 μl of nuclease-free water was added. The resulting reaction mixture was incubated at 16°C for 120 min and then at 70°C for 20 min. The reaction mixture was then diluted five-fold with nuclease-free water and stored at -30°C until RT-PCR analysis.

### Primer design and optimisation

Gene sequences were retrieved using the UCSC Genome Bioinformatics http://genome.ucsc.edu/ program, and primers sequences were designed using Primer3Plus http://www.bioinformatics.nl/cgi-bin/primer3plus/primer3plus.cgi. Specific interactions between primers and target genes were confirmed using either NCBI BLAST http://blast.ncbi.nlm.nih.gov/Blast.cgi) or BlastView (http://uswest.ensembl.org/index.html. The specificity of the expected RT-PCR products was determined based on melting curve analyses of reactions with glioma cDNA and human cDNA libraries. The product-specific melting curves showed only single peaks and no primer-dimer peaks or artefacts.

### Quantitative real-time reverse transcription-PCR

Quantitative PCR amplification assays were performed by a SYBR Green fluorescent assay using the ABI PRISM 7500 real-time PCR sequence detection system (Applied Biosystems, Foster City, CA, USA). Reactions were performed in a 96-well plate with 20-μl reaction solutions containing SYBR *Premix Ex Taq *II (10 μl) (Takara Bio., Inc., Japan), ROX reference dye II (0.4 μl), 10 μM forward and reverse primers (0.8 μl), 1 μl of cDNA template, and nuclease-free water (7 μl). Cycling conditions included an initial denaturation for 10 sec at 95°C, followed by 40 cycles of 5 sec at 95°C and 34 sec at 60°C. For determination of the reference gene, a standard curve was generated for each assay using seven serial dilutions of an amplified human brain cDNA library ranging from 20 ng to 20 fg.

The delta-delta Ct method was employed for the diagnostic assays. Ct values were calculated following the manufacturer's instructions (Applied Biosystems, Foster City, CA, USA), using *UBL5 *as the internal reference. The diagnostic genes fulfilled the criterion that the absolute value of the slope of the log input amount vs. *ΔCt *should be < 0.1.

### Data analysis

Thirty primers for the selected gene candidates and for the internal and negative controls were added in triplicate to 96-well plates, and the samples were measured using one plate per sample. The negative controls showed no detectable amplification or background levels of amplification (Ct ≥ 37, compared with 16 to 31 with sample DNAs). The mean and the standard deviation of differences of Ct values between duplicates were 0.060 and 0.086, respectively. Sequence detection software (Applied Biosystems) results were exported as tab-delimited text files and imported into Microsoft Excel for further analysis.

Statistical data processing was performed using Excel and SPSS, and Pearson's correlation coefficients (*r*) were computed for each cross-platform comparison. Progression-free survival was measured from the day of surgery to the time of the first event of progression or to the last day of follow-up, according to the Kaplan-Meier method. Curves were compared using the log-rank test.

## Results and Discussion

### Selection of the reference gene

We chose the delta-delta Ct method [9] for real-time PCR measurement rather than using calibration curves. Although the delta-delta Ct method has stricter requirements, it can substantially reduce the number of PCR reactions.

**Figure 1:**
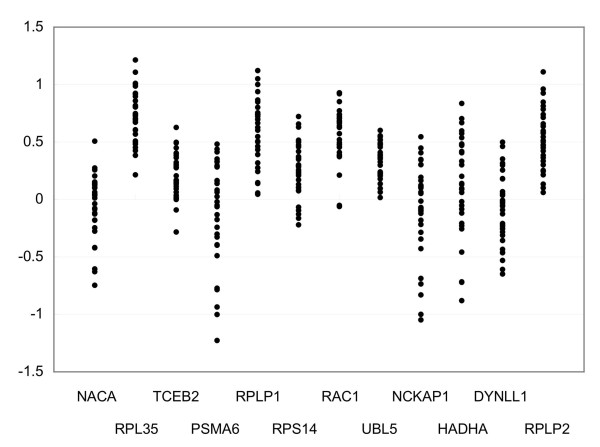
**Expression levels of control gene candidates**. Expression levels in 32 glioma tissues were measured and plotted for each gene.

The handling of gene expression data obtained by ATAC-PCR was similar to the handling of data from two-colour microarrays [12]. In both methods, the relative gene expression level compared to a control sample is measured and used for statistical analysis after data normalisation. In data normalisation of ATAC-PCR, each expression value was divided by the median of gene expression and then logarithmically converted. To choose the reference gene candidates whose expression was least changed between gliomas, we selected twelve genes exhibiting expression patterns that were most similar to the median gene expression pattern from 3,456 genes in the previous gene expression data matrix of 152 gliomas [5]. These twelve genes were expected to produce minimal variations in expression between glioma samples. To select the best reference gene, the expression levels of the twelve genes were measured in 32 glioma samples using real-time PCR. The results are shown in Figure 1. Gene expression values are influenced by the amount of mRNA and the random variation caused by biological and experimental factors [13]. Because variation in the amount of mRNA was common to all of the genes, the difference in measurement was primarily due to the latter. The measurement of *UBL5 *had the smallest variation; therefore, we selected it as the reference gene. Although the use of multiple reference genes is recommended by several reports [13,14], we chose a single reference gene for this case because the use of multiple reference genes would increase variations in measurement.

The first prognosis predictor developed for gliomas was based on the expression of 58 genes [5]. For the delta-delta Ct method, the amplification efficiency of a gene must be approximately equal to that of the reference gene. We performed real-time PCR amplification and fulfilled this criterion for 30 of the 58 genes. The original prognosis predictor classified gliomas into good and poor prognosis groups. The diagnostic scores calculated with the original 58 genes and the 30 genes chosen in this study had a high correlation (r = 0.95), and there was no difference between the classification results in the test set and those in the previous study [5]. Therefore, we decided to proceed with the 30 genes. A list of the genes and primer sequences is shown in Table 1.

**Table 1:** Primer sequences of the diagnostic genes

Gene Symbol	Forward	Reverse
IGFBP2	GCACATCCCCAACTGTGACA	TTCAGAGACATCTTGCACTGTTTG
VMP1	TGTCTTCTGTTGGGCTTGGAA	TGAGGCTATATGTGGACCCAGATA
MSN	GCCCCGGACTTCGTCTTC	AGGCCAAGATCCGCTTGTTA
TIMP1	CACAGACGGCCTTCTGCAAT	TGGTGTCCCCACGAACTTG
LGALS1	CTCCTGACGCTAAGAGCTTCGT	GAAGTGCAGGCACAGGTTGTT
CD63	CCCGAAAAACAACCACACTGC	GATGAGGAGGCTGAGGAGACC
NES	CAACAGCGACGGAGGTCTC	CCTCTACGCTCTCTTCTTTGAGT
CLIC1	TGTTCATGGTACTGTGGCTCAAG	GTCCGCCTTTTGGTGTCAAC
TNC	ACCACAATGGCAGATCCTTC	GCCTGCCTTCAAGATTTCTG
TAGLN2	CCTCTGGGAAGGAAAGAACATG	AGCCCACCCAGATTCATCAG
HES6	GACCAATGCCAGCCAGAG	GCAAGCCATCCATCAGAGG
VEGF	CCAAGGCCAGCACATAGGA	TCTTTGGTCTGCATTCACATTTG
VIM	TCCAAACTTTTCCTCCCTGAAC	GGGTATCAACCAGAGGGAGTGA
LDHA	CTGGGAGTTCACCCATTAAGCT	CAGGCACACTGGAATCTCCAT
RPIP8	CCCCCGTGGTCATCGA	GGTAGTCGTAGCTCTGCGTGAA
IFITM3	GGCTTCATAGCATTCGCCTACT	TCACGTCGCCAACCATCTT
PPIB	GGAGAGAAAGGATTTGGCTACAAA	CCTGGATCATGAAGTCCTTGATT
ALDOC	CGTCCGAACCATCCAGGAT	CCACACCCTTGTCAACCTTGAT
ZYX	CAGCAGCTAATGCAGGACATG	CAGAGTTCGTTGACAGCCACAT
UPAR	GTGTGTGGGTTAGACTTGTGCAA	AGGTAACGGCTTCGGGAATAG
LAMB2	CCACTGAAGGCGAGGTCATC	CCCGTAGGTTGGTGATCTTCAA
RTN1	CCGCATCTACAAGTCTGTTTTACAA	AAGCTCCAAGTAGGCCTTGAAAG
HMOX1	GGCAGAGAATGCTGAGTTCATG	AGGCCATCACCAGCTTGAAG
GM2A	GTCCCCCTGAGTTCTCCTCT	GCTCTTGGGCAGTGAGTAGG
S100A10	TGGAAAAGGAGTTCCCTGGAT	TACACTGGTCCAGGTCCTTCATT
BRSK2	GGAGGAGATGTCCAACCTGACA	AAGTTCCCAAACCAGGACTTCTT
MRCL3	AACAGAGATGGTTTCATCGACAAG	GTTGGATTCTTCCCCAATGAAG
GPX1	GCGGGGCAAGGTACTACTTA	CTCTTCGTTCTTGGCGTTCT
SOD2	AATCAGGATCCACTGCAAGGA	CGTGCTCCCACACATCAATC
RHOC	AATAAGAAGGACCTGAGGCAAGAC	ACGGGCTCCTGCTTCATCT
UBL5	AGCTGATTGCAGCCCAAACT	TCGTGTACCACTTCTTCAGGACAA

### Strategy for conversion

In our previous report of gene expression profiling of gliomas [5], we measured the relative expression levels against a control sample. Because the Ct value is inversely proportional to the amount of target nucleic acid present in the sample, the relative expression level of gene *i *of sample *x*, *er*_*i*_*(x)*, is described as follows:

$$er_{i}(x)={\left(1+E\right)}^{-(Ct_{i}(x)-Ct_{i}(c))}$$

Here, *Ct*_*i*_*(x) *and *Ct*_*i*_*(c) *are the Ct values of gene *i *of sample *x *and of the control sample, respectively. "1+*E*" represents the amplification efficiency of the real-time PCR, where 0 ≤ *E *≤ 1. The log-normalised gene expression, *en*_*i*_*(x)*, is obtained by the following conversion:

$$\begin{array}{c} en_{i}(x)=\log(er_{i}(x)/er_{UBL5}(x))\\ \;=-\log(1+E)*(Ct_{i}(x)-Ct_{UBL5}(x))\\ \;+\log(1+E)*(Ct_{i}(c)-Ct_{UBL5}(c)) \end{array}$$

Linear classifiers are most commonly used for molecular classification by gene expression profiles; an example is MammaPrint [2]. With a linear classifier, the diagnostic score is the sum of the normalised expression values multiplied by a coefficient determined from the learning data set. The diagnostic score of the prognosis predictor, the PC1 score, is described with Ct values as follows:

$$\begin{array}{c} PC1(x)=\sum_{i=1}^{n}a_{i}*en_{i}(x)\\ \;=-\log(1+E)*\sum_{i=1}^{n}a_{i}*(Ct_{i}(x)-Ct_{UBL5}(x))\\ \;+\log(1+E)*\sum_{i=1}^{n}a_{i}*(Ct_{i}(c)-Ct_{UBL5}(c)) \end{array}$$

Here, *PC1(x) *is the PC1 score of sample x. "*a*_*i*_" is a constant determined from the learning set in the previous study [5]. "*n*" is the number of diagnostic genes. *PC1(x) *is alternatively described as follows, defining *PC1*_*rt*_*(x*) as the PC1 score of sample x measured by real-time PCR.

$$PC1(x)=\beta_{1}*PC1_{rt}(x)+\beta_{0}$$

Here, *PC1*_*rt*_*(x*), *β*_1 _and *β*_0 _are as follows:

$$\begin{array}{c} PC1_{rt}(x)=\sum_{i=1}^{n}a_{i}*(Ct_{i}(x)-Ct_{UBL5}(x))\\ \beta_{1}=-\log(1+E)\\ \;\beta_{0}=\log(1+E)*\sum_{i=1}^{n}a_{i}*(Ct_{i}(c)-Ct_{UBL5}(c)) \end{array}$$

Because the *PC1(x) *value of the learning set was already determined, *β*_1 _and *β*_0 _can be determined by linear regression through measurement of *Ct*_*i*_*(x) *and *Ct*_*UBL5*_*(x) *of the corresponding samples. The conversion formula would then be validated with the test set. It should be noted that this method does not require the use of a control sample (i.e., measurement of *Ct*_*i*_*(c) *and *Ct*_*UBL5*_*(c)*).

### Construction of the prognosis predictor based on real-time PCR

**Figure 2:**
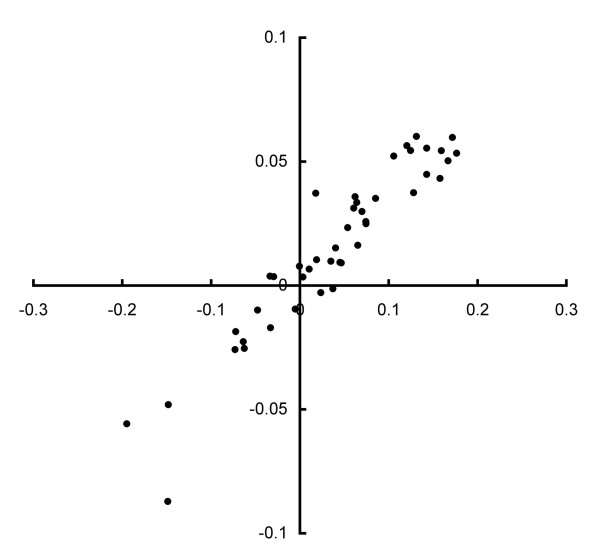
**The correlation of PC1 scores obtained using ATAC-PCR and real-time PCR**. Horizontal axis, PC1 score obtained with real-time PCR; vertical axis, PC1 score obtained with ATAC-PCR.

Using 44 samples from the learning set, we determined *PC1*_*rt*_*(x) *by measuring the Ct values. As expected, there was a high linear correlation between *PC1(x) *and *PC1*_*rt*_*(x) *(*r *= 0.94), as shown in Figure 2.

We then measured the correlation in individual gene expression (Table 2) between the ATAC-PCR data (log-normalised) and the *ΔCt *values (*ΔCt(x) = Ct*_*i*_*(x) *- *Ct*_*UBL5*_*(x)*). The correlation for individual genes was less robust than that for the PC1 score: the correlation coefficients ranged from 0.6 to 0.9. These results suggest that the PC1 score could eliminate errors in measurement through the weighted averaging of gene expression. Because three genes (*VMP1, TNC and RHOC*) exhibited no correlation, we eliminated them from the diagnostic gene set. Because ATAC-PCR uses a single gene-specific primer designed for the 3' end of the mRNA, it may be less specific than conventional PCR using two primers. The absence of correlation may be due to the amplification of different genetic fragments or splicing variants. The parameters of the conversion formula were determined by linear regression (*β*_1_, -0.37: *β*_0_, -0.002).

**Table 2:** Parameters for correlation between ATAC-PCR and real time PCR.

gene name	correlation coefficient	regression coefficient	intercept
IGFBP2	0.90	0.27	0.32
VMP1	0.04	0.05	2.87
MSN	0.81	0.36	1.00
TIMP1	0.92	0.30	-0.31
LGALS1	0.85	0.36	-0.56
CD63	0.51	0.20	-0.52
NES	0.69	0.26	0.69
CLIC1	0.86	0.43	0.34
TNC	0.04	-0.02	-0.63
TAGLN2	0.66	0.34	0.13
HES6	0.77	0.29	0.60
VEGF	0.78	0.25	-0.11
VIM	0.77	0.30	-0.52
LDHA	0.73	0.33	-0.12
RPIP8	0.81	0.26	0.71
IFITM3	0.85	0.38	-0.75
PPIB	0.60	0.29	-0.10
ALDOC	0.73	0.28	-0.09
ZYX	0.68	0.36	0.54
UPAR	0.84	0.36	1.48
LAMB2	0.43	0.23	0.62
RTN1	0.82	0.29	0.66
HMOX1	0.87	0.30	0.62
GM2A	0.51	0.24	0.62
S100A10	0.79	0.28	-0.18
BRSK2	0.68	0.22	1.21
MRCL3	0.73	0.30	0.38
GPX1	0.70	0.33	-0.41
SOD2	0.74	0.31	0.23
RHOC	0.11	-0.08	-0.06

Specific features of the expression of each gene may be obtained from the regression coefficient and intercept. Because the ATAC-PCR data were converted to a common logarithm during normalisation, the regression coefficient should be somewhere between zero and 0.30 (= log_10_2). In reality, the values ranged from 0.2 to 0.43, and ten genes demonstrated values exceeding 0.30. These results suggest a substantial degree of discrepancy between measurements obtained with ATAC-PCR and those determined using real-time PCR. The intercept indicates the general expression level of the gene; high intercept values indicate low levels of gene expression. With the exception of *VMP1*, the expression levels of the diagnostic genes were within two orders of magnitude of each other. The expression level of *UBL5 *was in the middle range of all of the diagnostic genes.

### Validation of the converted predictor

**Figure 3:**
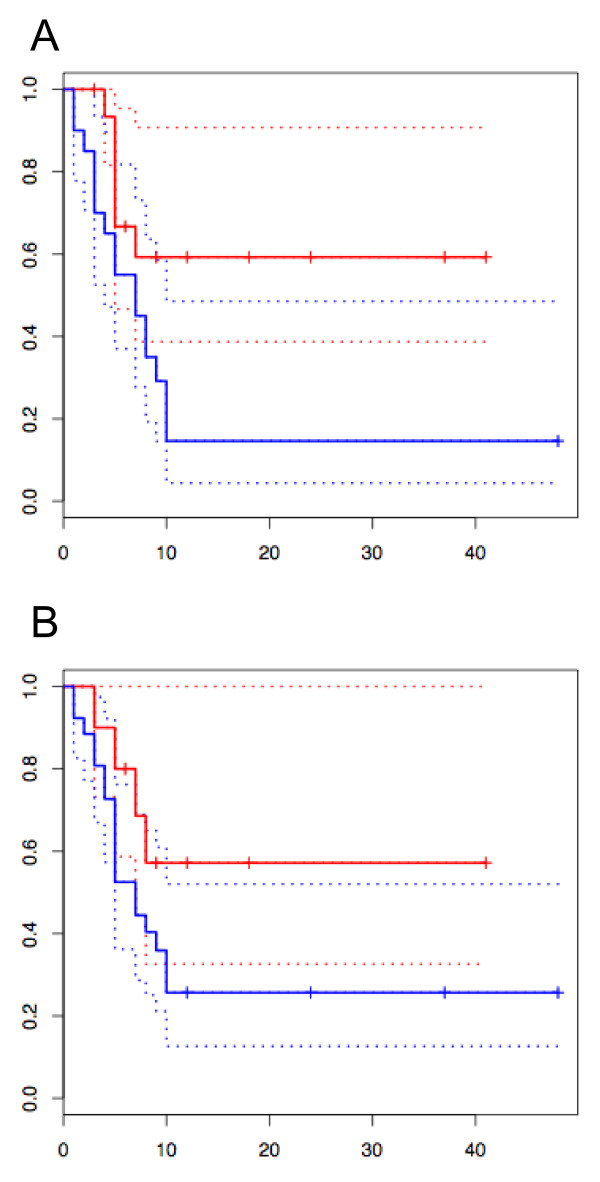
**Kaplan-Meier analysis of grade III and grade IV glioma patients stratified either by (A) PC1 scores from real-time PCR data or by (B) histopathological diagnosis**. Horizontal axis, month after diagnosis; vertical axis, progression-free survival probability. Blue lines, poor prognosis group (n = 20) (A) or grade IV (n = 26) (B); red lines, good prognosis group (n = 16) (A) or grade III (n = 10) (B). Log rank p-values were 0.023 (PC1 score) and 0.137 (histopathology). Dotted lines indicate 95% confidence intervals.

The converted predictor with 27 genes was validated with an additional sample set consisting of 16 samples from the previous test set [5] and 20 new samples. The samples were from anaplastic astrocytoma (grade III) or glioblastoma (grade IV). The PC1 score (*PC1(x)*) of each sample was calculated using *ΔCt *values measured using real-time PCR. The samples were classified into two prognosis groups with the threshold value set at zero, which was the threshold used in our previous study [5]. The performance of the classification was compared to conventional histopathological diagnosis. To have clinical utility, the predictor must have a classification ability superior to that of histopathological classification. The results of the Kaplan-Meier plot from the 36 samples revealed that the molecular classification was superior to histopathological diagnosis (log rank p-values, 0.023 and 0.137, respectively) (Figures 3A, B). The hazard ratio was 2.70 (95% confidence interval, 1.05-6.92) (p = 0.039) for molecular classification. No significant hazard ratio was obtained with histopathology (p = 0.16). We also noted that the classification results for the 16 samples from the original test set were the same as those previously obtained by ATAC-PCR. Thus, the new predictor based on real-time PCR is comparable to the previous predictor based on ATAC-PCR.

### Further considerations

In the delta-delta Ct method, the selection of the reference gene is the most important technical point. It has been frequently noted that housekeeping genes are not necessarily adequate for use as reference genes [14,15] because of their variable expression levels. Although it is possible to use a combination of housekeeping genes [14], a reference gene or a set of reference genes selected from the expression data matrix of the target tissues is more desirable because the measurement of other tissues is not performed in diagnostic practice. We selected a reference gene from a set of genes exhibiting expression patterns that were similar to the median gene expression pattern for the glioma data. Alternative methods to select reference genes should also be applicable to the conversion method described here [13,16].

In the present study, the original classifier was developed from gene expression data obtained by ATAC-PCR. Our conversion method is based on the linear correlation between gene expression profiling data and *ΔCt *values. A linear correlation was observed between normalised microarray data and *ΔCt *values regardless of the normalisation procedure [17]. Thus, our method should also be applicable to linear classifiers obtained using microarrays. As described above, the correlation between diagnostic scores is higher than that between individual genes. As demonstrated by diagnostic tests for breast cancer, the scores calculated from multiple gene expression correlate with the biology (malignancy) much better than individual gene expression, which includes noise of biological and experimental origin. The higher correlation of diagnostic scores between the two PCR techniques is not surprising. This result suggests that the conversion should be performed with the diagnostic score; it is not appropriate to perform the conversion at the level of individual gene expression.

It should be noted that validation experiments were performed only for the conversion process and that the predictor itself is in the preliminary stages of development and still needs analytical clinical validation and clinical utility studies. In particular, because the original predictor may also be applicable for the prognosis prediction of grade II gliomas [5], the future cohort should include a large number of grade II gliomas. In grade II and III glioma patients, the optimal timing of radiation therapy is still controversial [18,19]. Precise risk assessment, including the ability to predict possible malignant transformation, may be useful for timing decisions and is the most promising feature of the new classification scheme.

## Conclusions

We successfully converted a molecular classifier obtained by ATAC-PCR into a Ct value-based classifier. Our conversion procedure should also be applicable to linear classifiers developed from microarray data. Because errors in measurement are likely to be cancelled out during the calculation, the conversion of individual gene expression data is not an appropriate procedure. The predictor for gliomas is still in the preliminary stages of development and requires analytical clinical validation and clinical utility studies.

## Authors' contributions

KK conceived and designed the study. SK performed the experimental work following advice from KT and YK. Statistical analysis was done by KK, MS and MK. MS, NM, NH, SM and JT recruited the glioma patients and were responsible for the clinical aspects of the study. KK and SK wrote the manuscript. All authors have read and approved the manuscript.

## Pre-publication history

The pre-publication history for this paper can be accessed here:

http://www.biomedcentral.com/1755-8794/3/52/prepub

## References

[B1] Glas AM Floore A Delahaye LJ Witteveen AT Pover RC Bakx N Lahti-Domenici JS Bruinsma TJ Warmoes MO Bernards R Converting a breast cancer microarray signature into a high-throughput diagnostic test BMC Genomics 2006 7 278 10.1186/1471-2164-7-278 17074082 PMC1636049

[B2] van 't Veer LJ Dai H van de Vijver MJ He YD Hart AA Mao M Peterse HL van der Kooy K Marton MJ Witteveen AT Gene expression profiling predicts clinical outcome of breast cancer Nature 2002 415 530 536 10.1038/415530a 11823860

[B3] Paik S Development and clinical utility of a 21-gene recurrence score prognostic assay in patients with early breast cancer treated with tamoxifen Oncologist 2007 12 631 635 10.1634/theoncologist.12-6-631 17602054

[B4] Paik S Shak S Tang G Kim C Baker J Cronin M Baehner FL Walker MG Watson D Park T A multigene assay to predict recurrence of tamoxifen-treated, node-negative breast cancer N Engl J Med 2004 351 2817 2826 10.1056/NEJMoa041588 15591335

[B5] Shirahata M Oba S Iwao-Koizumi K Saito S Ueno N Oda M Hashimoto N Ishii S Takahashi JA Kato K Using gene expression profiling to identify a prognostic molecular spectrum in gliomas Cancer Sci 2009 100 165 172 10.1111/j.1349-7006.2008.01002.x 19038000 PMC11159015

[B6] Kato K Adaptor-tagged competitive PCR: a novel method for measuring relative gene expression Nucleic Acids Res 1997 25 4694 4696 10.1093/nar/25.22.4694 9358186 PMC147082

[B7] Kita-Matsuo H Yukinawa N Matoba R Saito S Oba S Ishii S Kato K Adaptor-tagged competitive polymerase chain reaction: amplification bias and quantified gene expression levels Anal Biochem 2005 339 15 28 10.1016/j.ab.2004.11.014 15766705

[B8] Coons SW Johnson PC Scheithauer BW Yates AJ Pearl DK Improving diagnostic accuracy and interobserver concordance in the classification and grading of primary gliomas Cancer 1997 79 1381 1393 10.1002/(SICI)1097-0142(19970401)79:7<1381::AID-CNCR16>3.0.CO;2-W 9083161

[B9] Livak KJ Schmittgen TD Analysis of relative gene expression data using real-time quantitative PCR and the 2(-Delta Delta C(T)) Method Methods 2001 25 402 408 10.1006/meth.2001.1262 11846609

[B10] Wang Y Barbacioru C Hyland F Xiao W Hunkapiller KL Blake J Chan F Gonzalez C Zhang L Samaha RR Large scale real-time PCR validation on gene expression measurements from two commercial long-oligonucleotide microarrays BMC Genomics 2006 7 59 10.1186/1471-2164-7-59 16551369 PMC1435885

[B11] Aoki T Takahashi JA Ueba T Oya N Hiraoka M Matsui K Fukui T Nakashima Y Ishikawa M Hashimoto N Phase II study of nimustine, carboplatin, vincristine, and interferon-beta with radiotherapy for glioblastoma multiforme: experience of the Kyoto Neuro-Oncology Group J Neurosurg 2006 105 385 391 10.3171/jns.2006.105.3.385 16961130

[B12] Schena M Shalon D Davis RW Brown PO Quantitative monitoring of gene expression patterns with a complementary DNA microarray Science 1995 270 467 470 10.1126/science.270.5235.467 7569999

[B13] Andersen CL Jensen JL Orntoft TF Normalization of real-time quantitative reverse transcription-PCR data: a model-based variance estimation approach to identify genes suited for normalization, applied to bladder and colon cancer data sets Cancer Res 2004 64 5245 5250 10.1158/0008-5472.CAN-04-0496 15289330

[B14] Vandesompele J De Preter K Pattyn F Poppe B Van Roy N De Paepe A Speleman F Accurate normalization of real-time quantitative RT-PCR data by geometric averaging of multiple internal control genes Genome Biol 2002 3 RESEARCH0034 10.1186/gb-2002-3-7-research0034 12184808 PMC126239

[B15] Guenin S Mauriat M Pelloux J Van Wuytswinkel O Bellini C Gutierrez L Normalization of qRT-PCR data: the necessity of adopting a systematic, experimental conditions-specific, validation of references J Exp Bot 2009 60 487 493 10.1093/jxb/ern305 19264760

[B16] Su LJ Chang CW Wu YC Chen KC Lin CJ Liang SC Lin CH Whang-Peng J Hsu SL Chen CH Huang CY Selection of DDX5 as a novel internal control for Q-RT-PCR from microarray data using a block bootstrap re-sampling scheme BMC Genomics 2007 8 140 10.1186/1471-2164-8-140 17540040 PMC1894975

[B17] Barbacioru CC Wang Y Canales RD Sun YA Keys DN Chan F Poulter KA Samaha RR Effect of various normalization methods on Applied Biosystems expression array system data BMC Bioinformatics 2006 7 533 10.1186/1471-2105-7-533 17173684 PMC1764432

[B18] van den Bent MJ Afra D de Witte O Ben Hassel M Schraub S Hoang-Xuan K Malmstrom PO Collette L Pierart M Mirimanoff R Karim AB Long-term efficacy of early versus delayed radiotherapy for low-grade astrocytoma and oligodendroglioma in adults: the EORTC 22845 randomised trial Lancet 2005 366 985 990 10.1016/S0140-6736(05)67070-5 16168780

[B19] Wick W Hartmann C Engel C Stoffels M Felsberg J Stockhammer F Sabel MC Koeppen S Ketter R Meyermann R NOA-04 randomized phase III trial of sequential radiochemotherapy of anaplastic glioma with procarbazine, lomustine, and vincristine or temozolomide J Clin Oncol 2009 27 5874 5880 10.1200/JCO.2009.23.6497 19901110

